# Glucocorticoid receptor regulates osteoblast differentiation in association with Map7d2 and Erk2 signaling

**DOI:** 10.3389/fimmu.2026.1967862

**Published:** 2026-10-29

**Authors:** Christoph Kölbl, Amelie Eckert, Sandra Nungeß, Sooyeon Lee, Jan Tuckermann, Anita Ignatius, Mubashir Ahmad

**Affiliations:** 1 Institute of Orthopedic Research and Biomechanics, Ulm University Medical Center, Ulm, Germany; 2 Institute of Molecular Endocrinology and Physiology, Ulm University, Ulm, Germany; 3 Integrated 3R Center Ulm (i3RU), Ulm University, Ulm, Germany; 4 German Center for Child and Adolescent Health (DZKJ), partner site Ulm, Ulm, Germany

**Keywords:** BMSCs, GR, MAP7D2, MAPK signaling, osteoblast differentiation, primary osteoblasts

## Abstract

The glucocorticoid receptor (GR) is an important regulator of skeletal homeostasis; however, the molecular mechanisms through which it regulates osteogenic differentiation remain incompletely understood. Here, we investigated the role of *GR* using two complementary primary murine osteogenic cell models, bone marrow stromal cells (BMSCs) and bone-derived primary osteoblasts, together with comparative transcriptomic analyses. *GR* depletion significantly impaired osteogenic differentiation in both cell preparations, as demonstrated by reduced alkaline phosphatase (Alp) activity and altered expression of osteogenic markers. RNA sequencing (RNA-seq) revealed extensive transcriptional changes following *GR* knockdown, with 3,530 differentially expressed genes (DEGs) in BMSCs and 4,657 in primary osteoblasts. Despite substantial differences in gene expression profiles of the two osteogenic cell models, pathway enrichment analyses revealed common biological processes, including MAPK signaling and cell migration. Comparative transcriptomic analysis further identified only 17 genes that were consistently regulated in the same direction in both cell preparations, among which *Map7d2* emerged as a previously unrecognized GR-responsive candidate. Combined depletion of *GR* and *Map7d2* partially restored cellular Alp activity and the expression of selected osteogenic markers compared with *GR* depletion alone. Western blot analysis further showed restoration of Erk2 phosphorylation and Alp protein expression following combined *GR* and *Map7d2* depletion, demonstrating an association between the partial rescue of selected osteogenic readouts and restoration of Erk2 signaling. Together, these findings identify *Map7d2* as a previously unrecognized GR-responsive candidate associated with Erk2 signaling and osteogenic differentiation. Our study reveals common molecular responses to *GR* depletion across two primary murine osteogenic cell models and provides new insights into the transcriptional and signaling networks underlying GR-regulated osteogenic differentiation.

## 1 Introduction

Bone is a dynamic tissue that undergoes continuous remodeling throughout life to maintain skeletal integrity, mineral homeostasis, and mechanical strength. This process depends on the tightly coordinated activities of bone-forming osteoblasts, bone-resorbing osteoclasts, and mechanosensitive osteocytes, whose balanced interactions preserve skeletal homeostasis. Disruption of this balance contributes to numerous skeletal disorders, including osteoporosis, inflammatory bone loss, and impaired fracture healing (1–4). Osteoblasts can originate from heterogeneous skeletal stem and progenitor cell (SSPC) populations residing in distinct skeletal niches, including the bone marrow and periosteum (5–9). Recent lineage-tracing and single-cell studies have revealed considerable heterogeneity among these osteoprogenitor populations and challenged the concept of a single, uniformly defined mesenchymal stem cell population giving rise to osteoblasts (8, 10). In the present study, the term bone marrow stromal cells (BMSCs) refers to the heterogeneous adherent stromal cell population obtained using established isolation and culture procedures and containing cells with osteogenic capacity (11, 12).

Osteoblast differentiation is orchestrated by complex transcriptional and signaling networks that integrate developmental, hormonal, and environmental signals. Lineage-defining transcription factors, including runt related transcription factor 2 (*Runx2*), Sp7 transcription factor 7 (*Sp7*), and members of the activator protein 1 (AP-1) family, act together with signaling pathways such as Wnt/β-catenin, bone morphogenetic protein (BMP)/transforming growth factor, β (TGF-β), Hedgehog, Notch, phosphoinositide 3-kinase (PI3K)/AKT, and mitogen-activated protein kinase (MAPK)/extracellular signal-regulated kinase (ERK) signaling, to regulate osteoblast commitment, differentiation, matrix production, and mineralization (13–17). Among the hormonal regulators of skeletal cells, the glucocorticoid receptor (GR), encoded by *Nr3c1*, is recognized as an important regulator of bone formation and skeletal homeostasis. GR belongs to the nuclear receptor superfamily and functions as a ligand-dependent transcriptional regulator of diverse biological processes, including cell proliferation, differentiation, metabolism, extracellular matrix organization, and inflammatory responses (18, 19). Substantial evidence indicates that glucocorticoids (GCs) and GR regulate osteoblastogenesis, osteoblast proliferation and differentiation, apoptosis of osteoblasts and osteocytes, Wnt and cytokine signaling, and bone formation (20–26). Genetic studies have further revealed direct and cell-specific functions of GR in osteoblast-lineage cells and established that GR signaling contributes substantially to the skeletal effects of GCs (22, 24, 27–29). In addition to systemic GC levels, local GC availability within bone is regulated by 11β-hydroxysteroid dehydrogenase type 1 (11β-HSD1), whose activity in osteoblasts can influence local GC action and susceptibility to GC-induced bone loss (30–32). GR signaling also contributes to skeletal regeneration, as global GR deletion impairs bone fracture healing and endochondral ossification (33). However, despite considerable progress in elucidating GC and GR action in skeletal cells, the molecular mechanisms through which GR regulates osteogenic differentiation remain incompletely understood.

Here, we investigated GR-dependent regulation of osteogenic differentiation using two independent primary murine osteogenic cell models: cultured BMSCs and primary osteoblasts derived from long bones. We treated them as independent osteogenic systems and examined the molecular responses to GR depletion shared between them. By combining functional analyses with comparative transcriptomics, we aimed to identify robust, shared GR-regulated pathways and GR-responsive candidates associated with osteogenic differentiation.

## 2 Materials and methods

### 2.1 Primary murine osteoblast and BMSC isolation and culture

Primary osteoblasts and bone marrow stromal cells (BMSCs) were isolated from the long bones of 12-week-old mice in accordance with local legislation and institutional requirements and with approval from the Regierungspräsidium Tübingen, Germany, as previously described (34). Briefly, the femora, tibiae, humeri, radii, and ulnae were harvested under sterile conditions and cleared of residual soft tissue. The bones were washed in phosphate-buffered saline (PBS; Gibco, Cat. 14190-094), and the epiphyses were manually removed. Bone marrow was collected by centrifugation at 12,500 rpm for 1 min at room temperature (RT). The resulting pellet was resuspended and transferred to T75 culture flasks containing BMSC growth medium composed of DMEM/F-12 (Gibco, Cat. 21331-020), 15% fetal bovine serum (FBS; Gibco, Cat. A5256701), 1% penicillin–streptomycin (Gibco, Cat. 15140-122), 1% L-glutamine (Gibco, Cat. 25030-024), and 50 µM β-mercaptoethanol (Gibco, Cat. 31350-010). After initial medium changes at 2 and 12 h, the medium was renewed every third day. Cells reached 70–80% confluence within approximately 2 weeks.

For primary osteoblast isolation, the diaphyses were cut into 1–2 mm fragments and digested with 1 mg/mL collagenase IV (Sigma-Aldrich, Cat. C6885) in α-MEM (Sigma-Aldrich, Cat. M4526) for 1 h at 37 °C in a shaking water bath. The bone fragments were then transferred to 56 cm² culture dishes containing complete growth medium (α-MEM supplemented with 15% FBS, 1% penicillin–streptomycin, and 1% L-glutamine). Cells were cultured at 37 °C in a humidified atmosphere containing 5% CO_2_ for 4 weeks, and adherent cells were harvested by trypsinization. Experiments were performed when cells reached approximately 80% confluence.

### 2.2 siRNA transfection and osteoblast differentiation

For siRNA-mediated knockdown followed by osteogenic induction (OI), BMSCs and primary osteoblasts were seeded at densities of 25,000 cells/cm² and 20,000 cells/cm², respectively. All siRNAs were obtained from Horizon Discovery (PerkinElmer) as four-sequence siGENOME SMARTpool preparations: siGENOME Non-Targeting Control siRNA Pool #2 (Dharmacon, Cat. D-001206-14-05), siGENOME mouse *GR* (Dharmacon, Cat. M-045970-01-0005), and siGENOME mouse *Map7d2* (Dharmacon, Cat. M-059254-01-0005). The component siRNA sequences are provided in **Table 1**. Cells were reverse transfected on day 0 with 0.125% Lipofectamine RNAiMAX (Thermo Fisher Scientific, Cat. 13778075) at a final total siRNA concentration of 20 nM, following a previously described protocol (35). Cells were allowed to adhere for 2 days before osteogenic induction was initiated. On days 2 and 5, the medium was replaced with osteogenic induction medium consisting of growth medium supplemented with 100 µg/mL (+)-sodium L-ascorbate (Sigma-Aldrich, Cat. A4034) and 5 mM β-glycerophosphate (Sigma-Aldrich, Cat. G5422). Endpoint analyses were performed on day 8, after 6 days of osteogenic induction.

**Table 1:** SMARTpool siRNA sequences targeting mouse genes used in this study.

Gene	Ensembl ID	siRNA target sequence(s)
*GR*	ENSMUSG00000024431	GAUCGAGCCUGAGGUGUUA UUACAAAGAUUGCAGGUAU GCCAAGAGUUAUUUGAUGA GCAUGUAUGACCAAUGUAA
*Map7d2*	ENSMUSG00000041020	GAGGAAAGGCUGCGCAUAG GAGCCAAGCUUCAGUAUGA CAACAUCAACCAUGAAUUU GCGCAUGGCUGACAAGUAU
Non-Targeting #2	-	UAGCGACUAAACACAUCAA UAAGGCUAUGAAGAGAUAC AUGUAUUGGCCUGUAUUAG AUGAACGUGAAUUGCUCAA

### 2.3 Cell viability assay

Cell viability was assessed using the PrestoBlue Cell Viability reagent (Thermo Fisher Scientific, Cat. A13261) according to the manufacturer’s instructions. Briefly, in 24-well plates, cells were incubated with a mixture of PrestoBlue reagent and culture medium (1:10 ratio). In parallel, a blank control consisting of culture medium and reagent (1:10) was prepared in a 96-well plate. Cells were incubated at 37 °C in a humidified atmosphere with 5% CO_2_ for approximately 1 h. Subsequently, 100 µL aliquots were transferred to a 96-well plate, and absorbance was measured at 570 nm using a Spark Multimode Microplate Reader (TECAN, Cat. 30086376), with values normalized to blank controls.

### 2.4 Quantitative and qualitative alkaline phosphatase staining

Alkaline phosphatase (Alp) staining was assessed qualitatively and quantitatively using established protocols (35, 36). For qualitative staining, cells were washed with PBS and fixed for 10 min at RT with 4% paraformaldehyde (PFA) (Carl Roth, Cat. 0335.1) in PBS. Subsequently, a naphthol-based Alp staining solution (Sigma-Aldrich, Cat. 86R-1KT) was applied according to the manufacturer’s instructions, and cells were incubated in the dark at RT for 2 h. Stained cells were imaged using a VHX digital microscope (Keyence, Cat. VHX-7000).

For quantitative Alp activity analysis, cells were seeded in 24-well plates and cultured in OI medium. After assessment of cell viability, cells were washed with PBS and Alp activity was measured using the Amplite colorimetric Alp assay kit (Biomol, Cat. ABD-11950) following the manufacturer’s protocol. Briefly, cells were incubated with p-nitrophenyl phosphate (pNPP) substrate at 37 °C for 1 h, and the optical density (OD) was measured at 405 nm using a Spark Multimode Microplate Reader. Alp activity was normalized to cell viability, and the results were expressed as percentage relative to control (set at 100%).

### 2.5 RNA isolation, cDNA synthesis, and quantitative real-time PCR

Total RNA was isolated from cultured cells using the RNeasy Mini Kit (Qiagen, Cat. 75142) according to the manufacturer’s instructions. For cDNA synthesis, 1 µg of total RNA was reverse-transcribed using the Omniscript Reverse Transcriptase Kit (Qiagen, Cat. 205113). qRT-PCR was performed using the QuantStudio 3 Real-Time PCR System (Applied Biosystems). Amplification specificity was confirmed by melt-curve analysis, and primer efficiency was validated using standard curves. Relative gene expression was calculated using the 2^-ΔΔCt^ method, with beta-2 microglobulin (*B2m*) used as the sole reference gene for normalization in all qRT-PCR experiments. Primer sequences are listed in **Table 2**.

**Table 2:** Mouse primer sequences used for qRT-PCR in this study.

Gene symbol	Forward primer (5’ -> 3’)	Reverse primer (5’ -> 3’)
*Alpl*	GCT GAT CAT TCC CAC GTT TT	CTG GGC CTG GTA GTT GTT GT
*B2m*	ATA CGC CTG CAG AGT TAA GCA	TCA CAT GTC TCG ATC CCA GT
*Bglap*	GCG CTC TGT CTC TCT GAC CT	ACC TTA TTG CCC TCC TGC TT
*Col1a1*	GCT GCA TAC ACA ATG GCC TA	TCA AGC ATA CCT CGG GTT TC
*Map7d2*	TGC AAA CGA AGA AAC ACC AA	CTT ATC TGC TAC ATA CTT CTC GG
*GR (Nr3c1)*	GGC CGC TCA GTG TTT TCT AA	GCA GAG TTT GGG AGG TGG T
*Runx2*	CCA CCA CTC ACT ACC ACA CG	CAC TCT GGC TTT GGG AAG AG
*Sp7*	CCT TAA CCC AGC TCC CTA CC	ACC GCC TTG GGC TTA TAG AC

### 2.6 RNA sequencing and bioinformatic analyses

For transcriptome analysis of *in vitro* samples, total RNA was isolated using the RNeasy Mini Kit (Qiagen, Cat. 75142), as described above. RNA concentration and quality were assessed using the 2100 Bioanalyzer with the RNA 6000 Nano Kit (Agilent Technologies). Only samples with an A_260_/A_280_ ratio of 1.9–2.0 were used. The BMSC dataset comprised three independent biological replicates per group, whereas the primary osteoblast dataset comprised four independent biological replicates per group. Library preparation and RNA sequencing were performed by Novogene using the Illumina HiSeq 2500 platform (Illumina). Raw sequencing reads were processed using fastp to remove adapter sequences and low-quality reads. High-quality reads were aligned to the mouse reference genome (GRCm38.p6, Ensembl) using STAR, and gene-level raw integer counts were generated using featureCounts.

The raw count matrix was used as input for DESeq2 in RStudio. DESeq2 normalization was based on sample-specific size factors estimated using the median-of-ratios method. Variance-stabilized count data were used for principal component analysis (PCA) with the pcaExplorer package (37) to assess sample variability and clustering. Differentially expressed genes (DEGs) were defined as those with a log_2_ fold change (log_2_FC) ≥ 1 or ≤ -1 and a false discovery rate (FDR) < 0.01. DEGs were visualized using volcano plots generated with the EnhancedVolcano package (38), and hierarchical clustering was performed using pheatmap. Gene Ontology enrichment analysis was performed using Enrichr (39), with all DEGs meeting these criteria as input and the whole mouse genome as the background gene set. Adjusted p-values reported by Enrichr were calculated using the Benjamini–Hochberg method. Enriched terms were visualized using Metascape (40). In the enrichment-network visualization, “common genes” were genes assigned to two or more displayed enriched terms, whereas “unique genes” were assigned to only one displayed term. These designations were not used as additional DEG-filtering criteria.

### 2.7 Protein isolation, quantification, and western blotting

For protein isolation, cells were harvested by manually scraping the monolayer and transferring the suspension into reaction tubes. Following centrifugation at 14,000 rpm at 4 °C for 5 min, the supernatant was discarded and total cellular protein was extracted from the cell pellet using radioimmunoprecipitation assay (RIPA) lysis buffer. Protein concentration was determined using Pierce BCA protein assay kit (Thermo Fisher Scientific, Cat. 23225) according to the manufacturer’s instructions. For immunoblotting, equal amounts of protein (25 µg per sample) were separated by sodium dodecyl sulfate-polyacrylamide gel electrophoresis (SDS-PAGE) and transferred onto nitrocellulose membranes as previously described (35). Membranes were incubated with primary antibodies against phospho-p44/42 MAPK (Erk1/2, Thr202/Tyr204; Cell Signaling Technology, Cat. 9101), total p44/42 MAPK (Erk1/2; Cell Signaling Technology, Cat. 9102), and alkaline phosphatase/Alp (R&D Systems, Cat. AF2910). α-Tubulin (Cell Signaling Technology, Cat. 2144) and β-actin (13E5; Cell Signaling Technology, Cat. 4970) were used as loading controls. Bound primary antibodies were detected using horseradish peroxidase (HRP)-conjugated secondary antibodies (anti-mouse IgG (Cell Signaling Technology, Cat. 7076), anti-rabbit IgG (Cell Signaling Technology, Cat. 7074), and anti-goat IgG (Thermo Fisher Scientific, Cat. 31402). Protein bands were visualized using the Immobilon Forte Western HRP substrate (Millipore, Cat. WBLUF0500) and imaged by chemiluminescence using the ChemiDocTM MP Imaging System (Bio-Rad, Cat. 734BR4566). Band intensities were quantified using ImageJ (41). Erk phosphorylation was expressed as the ratio of phosphorylated to corresponding total protein (p-Erk1/Erk1 and p-Erk2/Erk2). Representative western blot images present descriptive densitometry from two independent biological experiments (n=2 per group); given the limited number of biological replicates, no inferential statistical analysis was performed for these measurements.

### 2.8 Statistical analysis

The number of independent biological replicates per experimental group is indicated as *n* in the corresponding figure legends. Data are presented as scatter plots with the mean ± standard deviation (SD). Comparisons between two groups were performed using an unpaired two-tailed Student’s *t*-test. Experiments involving more than two groups were analyzed using one-way ANOVA followed by Holm–Sidak’s multiple-comparisons test. A *p*-value < 0.05 was considered statistically significant. For representative western blot images measurements from two independent biological experiments (n=2 per group) were analyzed descriptively and were not subjected to inferential statistical analysis because of the limited number of biological replicates.

## 3 Results

### 3.1 *GR* depletion impairs osteogenic differentiation and alters transcriptional programs in murine BMSCs

To investigate the role of *GR* in osteogenic differentiation, BMSCs were reverse-transfected with *GR*-specific siRNA (si*GR*) or non-targeting control siRNA (si*NT*) before osteogenic induction (**Figure 1A**). *GR* depletion significantly increased cell viability compared with si*NT* control cells (**Figure 1B**). In contrast, cellular alkaline phosphatase (Alp) activity was markedly reduced following *GR* knockdown (**Figure 1C**). Consistently, qualitative Alp staining demonstrated impaired osteogenic differentiation in si*GR*-treated BMSCs compared with si*NT* controls (**Figure 1D**). Efficient *GR* knockdown was confirmed by qRT-PCR, demonstrating a significant reduction in *GR* mRNA expression (**Figure 1E**). While runt related transcription factor 2 (*Runx2*) expression remained unchanged (**Figure 1F**), *GR* depletion significantly reduced the expression of the osteogenic transcription factor Sp7 transcription factor 7 (*Sp7*) and the early osteoblast marker alkaline phosphatase (*Alpl*) (**Figures 1G, H**). In contrast, collagen, type I, alpha 1 (*Col1a1*) and bone gamma carboxyglutamate protein (*Bglap*) expression was not significantly altered (**Figures 1I, J**). These findings indicate that *GR* is required for efficient osteogenic differentiation of murine BMSCs.

**Figure 1:**
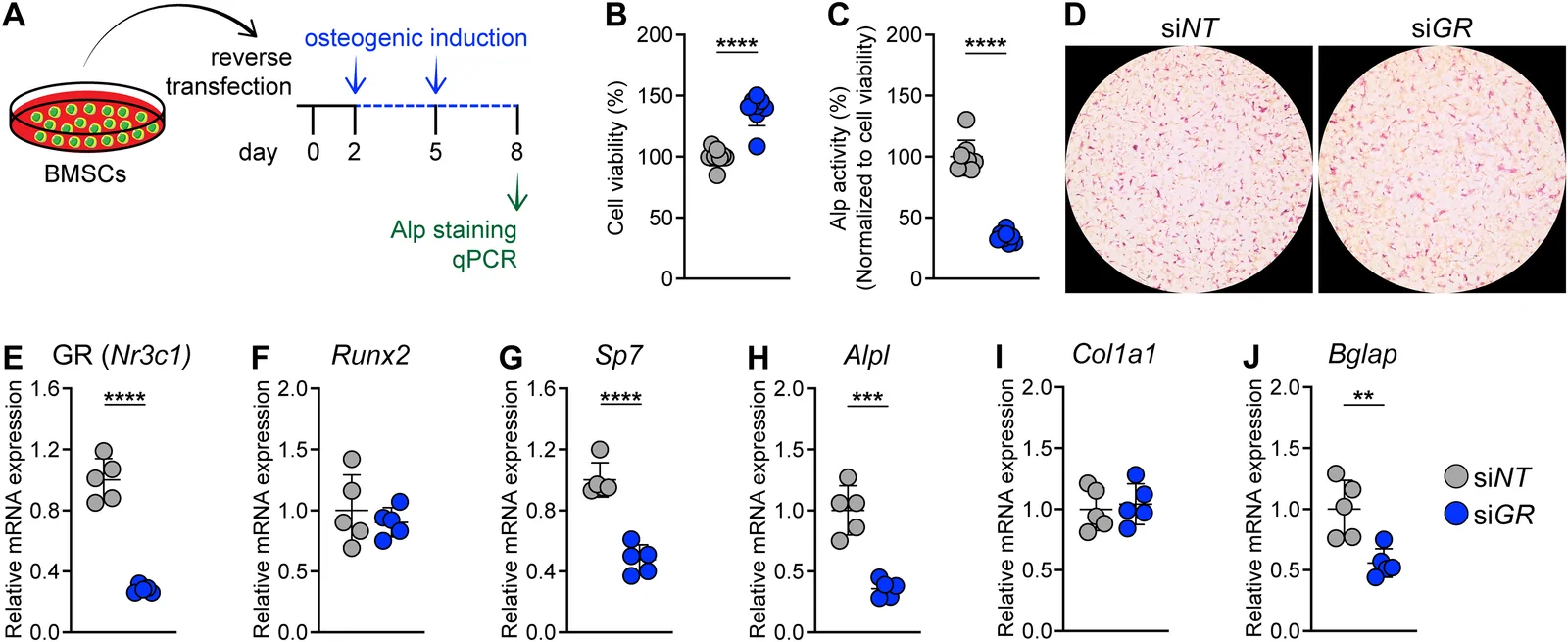
*GR* depletion impairs osteogenic differentiation of BMSCs. **(A)** Schematic overview of siRNA-mediated *GR* knockdown in BMSCs. **(B)** Cell viability and **(C)** quantitative alkaline phosphatase (Alp) activity in cells transfected with non-targeting control siRNA (si*NT*) or *GR*-specific siRNA (si*GR*) at day 8 post-transfection (n=8/group). **(D)** Representative qualitative Alp staining at day 8 post-transfection (n=8/group). **(E)** Expression of GR (*Nr3c1*) and **(F–J)** osteoblast-specific marker genes (*Runx2*, *Sp7*, *Alpl*, *Col1a1*, and *Bglap*) at day 8 post-transfection (n=5/group). Data are presented as mean ± SD. Statistical significance was determined using an unpaired two-tailed Student’s *t*-test. **p<0.01; ***p<0.001, ****p<0.0001.

To further define the transcriptional programs regulated by *GR* during osteogenic differentiation, RNA sequencing (RNA-seq) was performed in si*GR*- and si*NT*-transfected BMSCs following osteogenic induction (**Figure 2A**). Principal component analysis (PCA) revealed a clear separation between si*GR* and si*NT* samples, with the first principal component accounting for 94.61% of the variance, indicating profound transcriptomic alterations following *GR* depletion (**Figure 2B**). Differential gene expression analysis identified 3,530 significantly altered genes, of which 2,110 were upregulated and 1,420 were downregulated in *GR*-depleted BMSCs (**Figure 2C**; **Supplementary Table 1**). Pathway enrichment analysis of differentially expressed genes (DEGs) revealed a strong enrichment of pathways associated with extracellular structure organization, external encapsulating structure, and extracellular matrix organization (**Figure 2D**). Additional enriched pathways included positive regulation of cell population proliferation, regulation of cell migration, skeletal system development, and positive regulation of the MAPK cascade (**Figure 2D**). Together, these data demonstrate that *GR* depletion not only impairs osteogenic differentiation but also induces extensive transcriptional changes of genes encoding extracellular matrix, skeletal system development, and MAPK-associated pathways in murine BMSCs.

**Figure 2:**
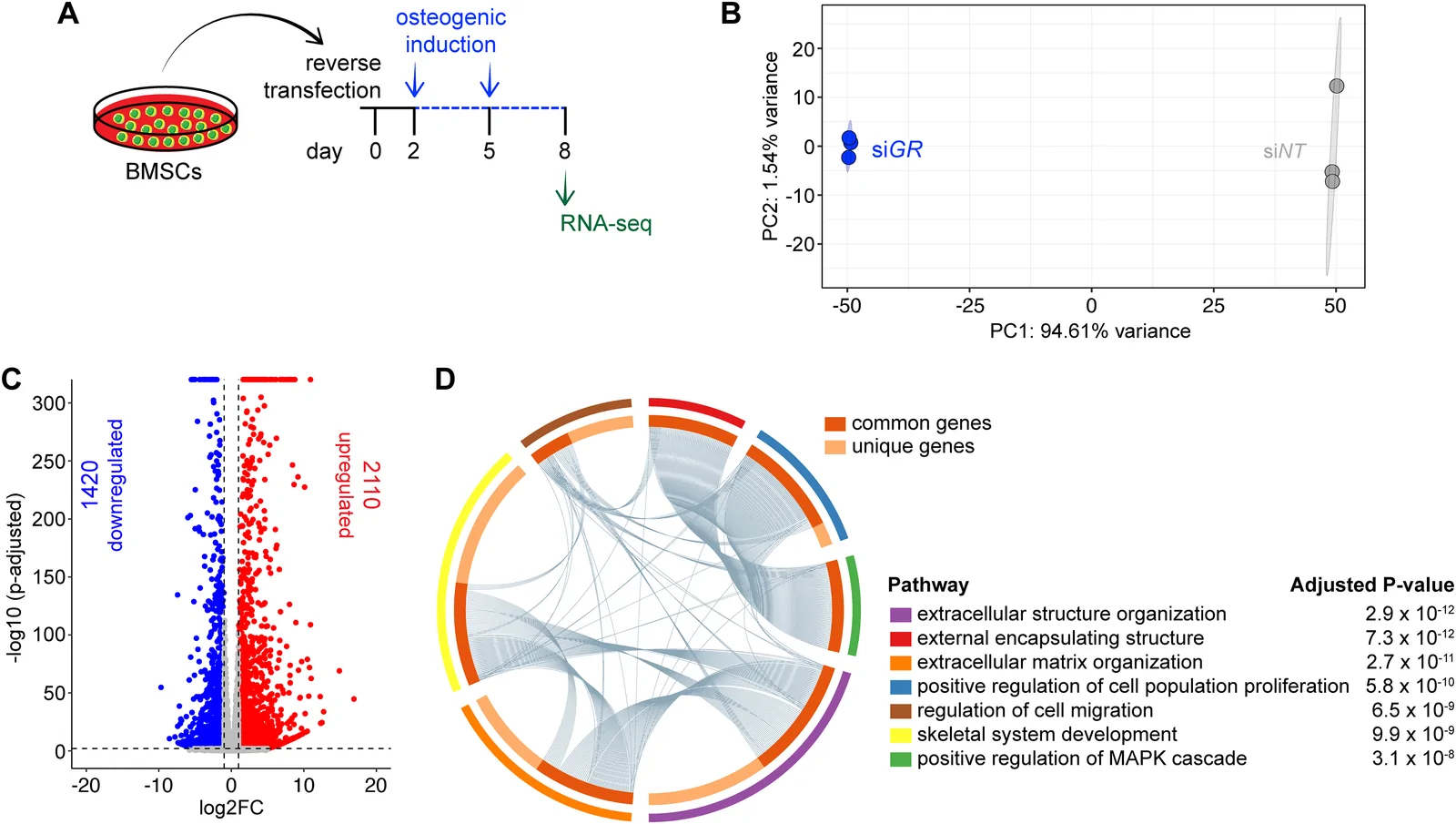
*GR* depletion induces extensive transcriptional reprogramming in BMSCs. **(A)** Schematic overview of siRNA-mediated *GR* knockdown and RNA sequencing (RNA-seq) in BMSCs. **(B)** Principal component analysis (PCA) of transcriptomic profiles of BMSCs transfected with si*NT* or si*GR* (n=3/group). **(C)** Volcano plot showing significantly downregulated (blue) and upregulated (red) differentially expressed genes (DEGs). **(D)** Pathway enrichment analysis of DEGs using Enrichr showing seven highly enriched biological pathways. Adjusted *p*-values correspond to the multiple-testing-adjusted *p*-values reported by Enrichr. The circular plot illustrates genes shared among enriched pathways, with blue connecting lines indicating genes occurring in more than one pathway.

### 3.2 *GR* depletion impairs osteoblast differentiation and alters transcriptional programs in primary murine osteoblasts

To determine the role of *GR* in osteogenic differentiation of primary osteoblasts, primary murine osteoblasts were isolated from long bones, reverse-transfected with si*GR* or si*NT*, and subjected to osteogenic induction (**Figure 3A**). *GR* depletion significantly reduced cell viability compared with si*NT* control cells (**Figure 3B**). Cellular Alp activity was also markedly reduced following *GR* knockdown (**Figure 3C**). Consistently, qualitative Alp staining demonstrated impaired osteogenic differentiation in si*GR*-treated primary osteoblasts compared with si*NT* controls (**Figure 3D**). Efficient *GR* knockdown was confirmed by qRT-PCR, demonstrating a significant reduction in *GR* mRNA expression (**Figure 3E**). *GR* depletion significantly reduced the expression of *Runx2*, *Sp7*, *Alpl*, and *Col1a1* (**Figures 3F–I**), whereas *Bglap* expression remained unchanged (**Figure 3J**). These findings indicate that *GR* is required for efficient osteogenic differentiation of primary murine osteoblasts.

**Figure 3:**
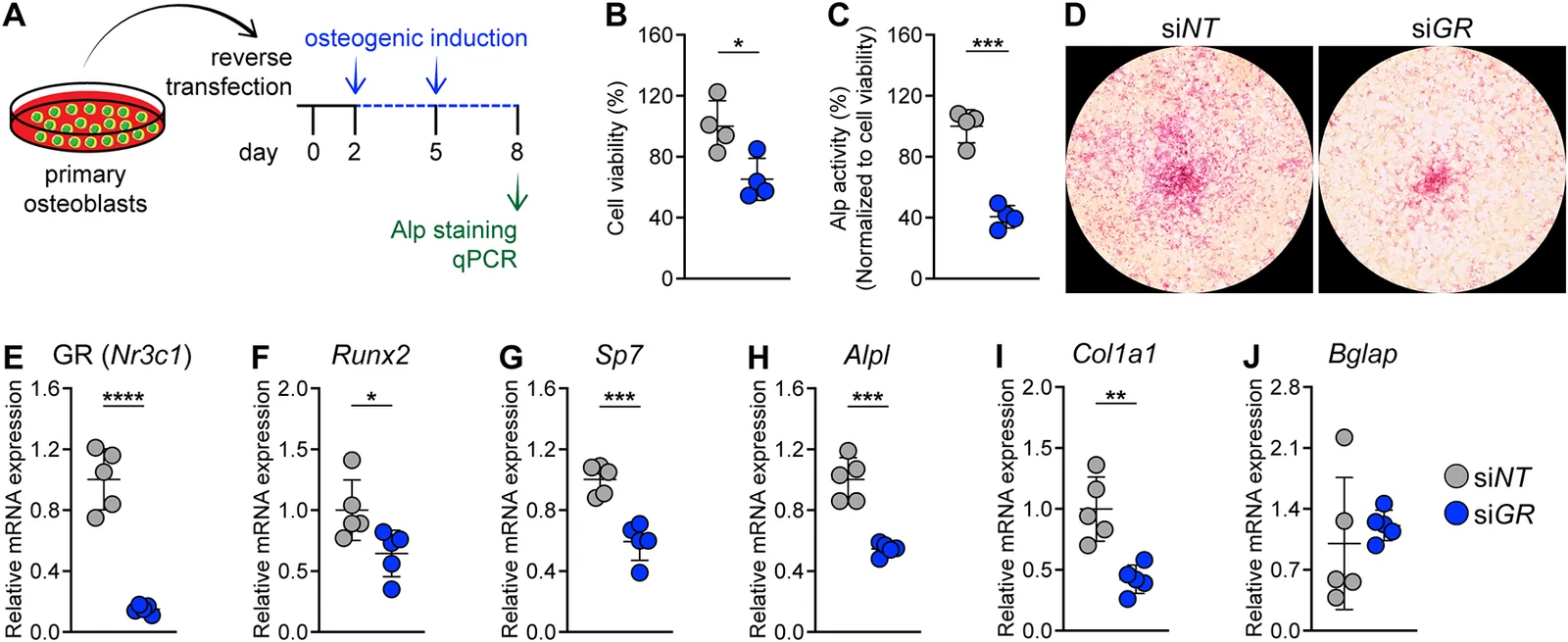
*GR* depletion impairs osteogenic differentiation in primary osteoblasts. **(A)** Schematic overview of siRNA-mediated *GR* knockdown in primary osteoblasts. **(B)** Cell viability and **(C)** quantitative Alp activity in cells transfected with si*NT* or si*GR* at day 8 post-transfection (n=4/group). **(D)** Representative qualitative Alp staining at day 8 post-transfection (n=4/group). **(E)** Expression of GR (*Nr3c1*) and **(F–J)** osteoblast-specific marker genes (*Runx2*, *Sp7*, *Alpl*, *Col1a1*, and *Bglap*) at day 8 post-transfection (n=5/group). Data are presented as mean ± SD. Statistical significance was determined using an unpaired two-tailed Student’s *t*-test. *p<0.05, **p<0.01, ***p<0.001, ****p<0.0001.

To further characterize the transcriptional programs regulated by *GR* in primary osteoblasts, RNA-seq was performed in si*GR*- and si*NT*-transfected cells following osteogenic induction (**Figure 4A**). PCA revealed a clear separation between si*GR* and si*NT* samples, with the first principal component accounting for 92.67% of the variance, indicating extensive transcriptomic alterations following *GR* depletion (**Figure 4B**). Differential gene expression analysis identified 4,657 significantly altered genes, of which 2,004 were upregulated and 2,653 were downregulated in *GR*-depleted primary osteoblasts (**Figure 4C**; **Supplementary Table 2**). Pathway enrichment analysis of DEGs revealed significant enrichment of pathways associated with positive regulation of the MAPK cascade, positive regulation of PI3K/PKB signal transduction, and regulation of cell migration (**Figure 4D**). Additional enriched pathways included positive regulation of cell migration, regulation of the MAPK cascade, regulation of the ERK1 and ERK2 cascade, and positive regulation of cell motility (**Figure 4D**). Collectively, these data demonstrate that *GR* depletion impairs osteogenic differentiation and induces extensive transcriptional reprogramming of MAPK-, ERK-, and PI3K/PKB-associated signaling pathways in primary murine osteoblasts.

**Figure 4:**
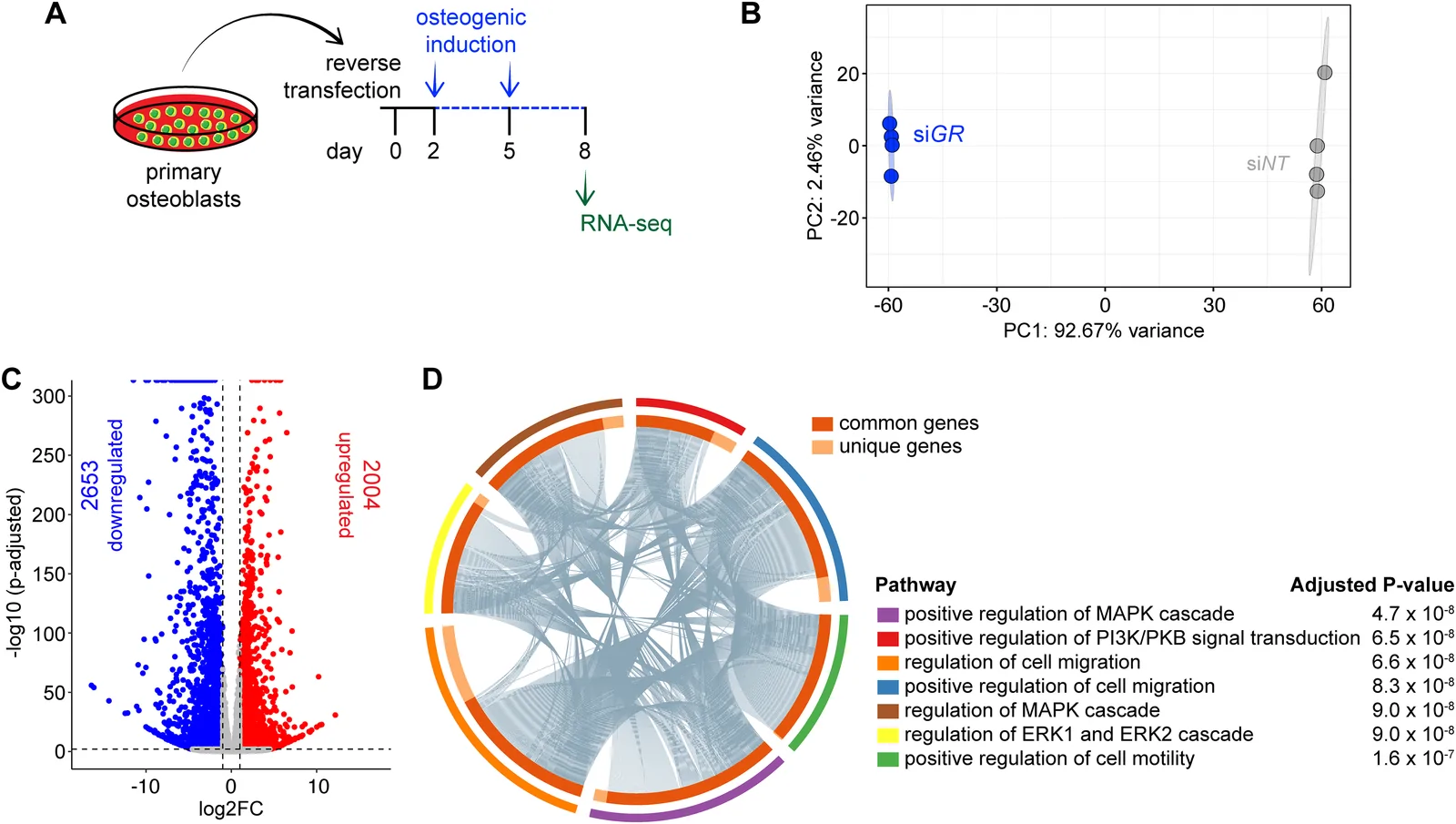
*GR* depletion induces extensive transcriptional reprogramming in primary osteoblasts. **(A)** Schematic overview of siRNA-mediated *GR* knockdown and RNA-seq in primary osteoblasts. **(B)** PCA of transcriptomic profiles of primary osteoblasts transfected with si*NT* or si*GR* (n=4/group). **(C)** Volcano plot showing significantly downregulated (blue) and upregulated (red) DEGs. **(D)** Pathway enrichment analysis of DEGs using Enrichr showing seven highly enriched biological pathways. Adjusted *p*-values correspond to the multiple-testing-adjusted *p*-values reported by Enrichr. The circular plot illustrates genes shared among enriched pathways, with blue connecting lines indicating genes occurring in more than one pathway.

### 3.3 Comparative transcriptomic analysis identifies common *GR*-regulated genes in BMSCs and primary osteoblasts

To identify shared transcriptional responses to *GR* depletion in BMSCs and primary osteoblasts, DEGs from the two RNA-seq datasets were compared. Of the 3,530 DEGs identified in BMSCs and the 4,657 DEGs identified in primary osteoblasts, 2,728 were differentially expressed in both datasets (**Figure 5A**). Of these overlapping DEGs, 2,711 were regulated in opposite directions, whereas only 17 were regulated concordantly (**Figure 5A**). These 17 genes therefore represented a small set of *GR*-responsive candidates with consistent directional regulation across both primary osteogenic cell models.

**Figure 5:**
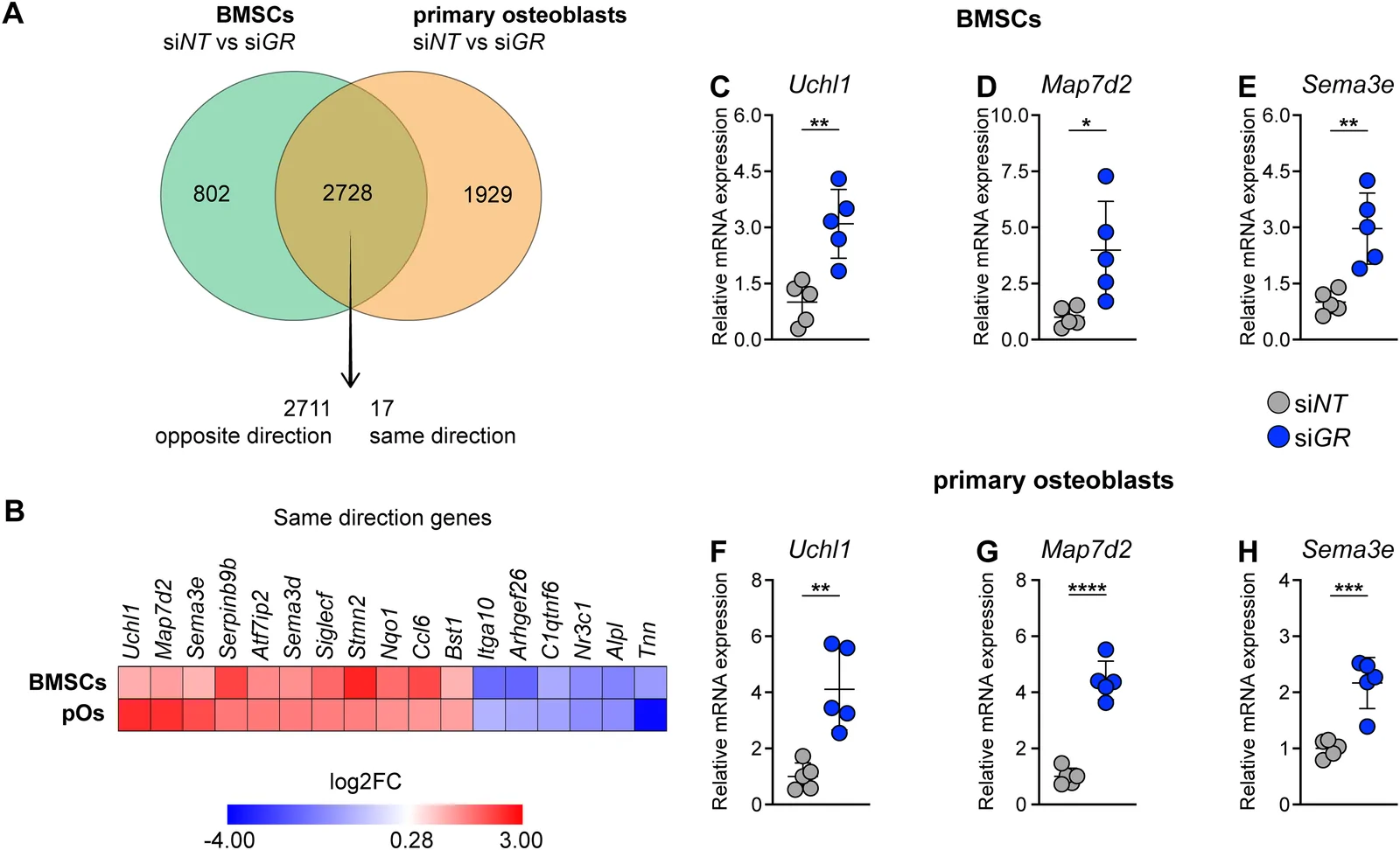
Comparative transcriptomic analysis identifies common *GR*-regulated genes in BMSCs and primary osteoblasts. **(A)** Venn Diagram illustrating the overlap and directionality of DEGs identified following *GR* depletion in BMSCs and primary osteoblasts (pOs). **(B)** Comparison of log2 fold changes of genes showing concordant regulation in both cell types. **(C–E)** Expression of *Uchl1, Map7d2, and Sema3e* in BMSCs (n=5/group) **(F–H)** and primary osteoblasts following *GR* depletion (n=5/group). Data are presented as mean ± SD. Statistical significance was determined using an unpaired two-tailed Student’s *t*-test. *p<0.05, **p<0.01, ***p<0.001, ****p<0.0001.

Visualization of the log2 fold changes for these 17 genes demonstrated concordant up- or downregulation following *GR* depletion in both cell populations (**Figure 5B**). Among the concordantly upregulated genes, ubiquitin C-terminal hydrolase L1 (*Uchl1*), MAP7 domain containing 2 (*Map7d2*), and sema domain, immunoglobulin domain (Ig), short basic domain, secreted, (semaphorin) 3E (*Sema3e*) were selected as robust candidates for independent qRT-PCR validation. qRT-PCR confirmed significantly increased expression of *Uchl1*, *Map7d2*, and *Sema3e* following *GR* depletion in both BMSCs and primary osteoblasts (**Figures 5C–H**), consistent with the RNA-seq results. *Map7d2* was prioritized for functional investigation because its role in osteogenic differentiation and skeletal biology remains poorly characterized.

### 3.4 Combined *GR* and *Map7d2* depletion partially restores osteogenic differentiation

To investigate whether *Map7d2* is associated with the impaired osteogenic differentiation observed following *GR* depletion, BMSCs were transfected with siRNAs targeting *GR*, *Map7d2*, or both simultaneously, followed by osteogenic induction (**Figure 6A**). Efficient knockdown of both *GR* and *Map7d2* was confirmed by qRT-PCR in the respective single-knockdown and co-transfection groups (**Figures 6B, C**). Silencing of *GR* significantly increased cell viability while reducing cellular Alp activity. In contrast, *Map7d2* knockdown did not affect cell viability but unexpectedly also reduced cellular Alp activity. Notably, simultaneous knockdown of *GR* and *Map7d2* partially restored Alp activity compared with *siGR*-transfected cells (**Figures 6D, E**). At the gene-expression level, *GR* depletion significantly reduced *Runx2*, *Sp7*, *Alpl*, *Col1a1*, and *Bglap*, whereas *Map7d2* depletion alone increased *Alpl* and *Bglap* (**Figures 6F–J**). The increase in these selected osteogenic transcripts following *Map7d2* depletion did not correspond to increased cellular Alp activity and therefore did not indicate enhanced osteogenic differentiation. Co-depletion of *GR* and *Map7d2* significantly restored *Sp7*, *Alpl*, and *Bglap* expression relative to *GR* depletion alone, whereas *Runx2* and *Col1a1* were not significantly restored (**Figures 6F–J**). These findings indicate a partial and marker-specific rescue of the effects of *GR* depletion. Collectively, these functional data demonstrate that combined *GR* and *Map7d2* depletion partially restores selected osteogenic readouts compared with *GR* depletion alone. However, the incomplete rescue and the discordance between osteogenic gene expression and cellular Alp activity indicate a complex relationship between *Map7d2* and the osteogenic phenotype following *GR* depletion. Moreover, these experiments do not establish whether *Map7d2* is a direct transcriptional target of GR.

**Figure 6:**
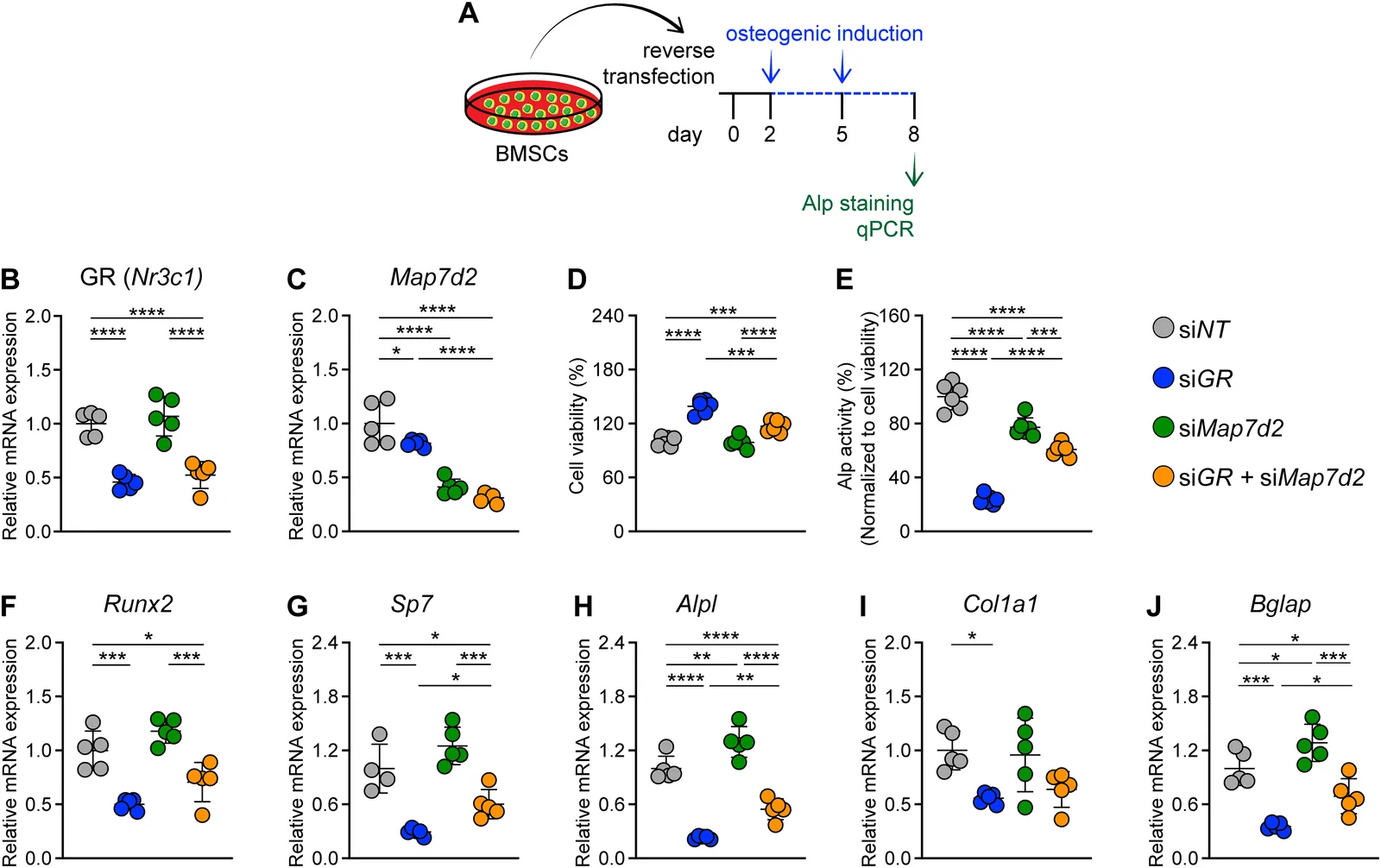
*Map7d2* contributes to the impaired osteogenic differentiation induced by *GR* depletion. **(A)** Schematic overview of siRNA-mediated knockdown of *GR* and *Map7d2*, individually or in combination, in BMSCs. **(B, C)** Expression of GR (*Nr3c1*) and *Map7d2* at day 8 post-transfection (n=5/group). **(D)** Cell viability and **(E)** quantitative Alp activity following *GR*, and/or *Map7d2* depletion (n=6/group). **(F–J)** Expression of osteoblast-specific marker genes (*Runx2*, *Sp7*, *Alpl*, *Col1a1*, and *Bglap*) at day 8 post-transfection (n=5/group). Data are presented as mean ± SD. Statistical significance was determined using one-way ANOVA followed by Holm-Sidak’s multiple-comparisons test. *p<0.05, **p<0.01, ***p<0.001, ****p<0.0001.

### 3.5 *Map7d2* is associated with altered Erk2 signaling in *GR*-depleted BMSCs

To examine whether the partial rescue observed following combined *GR* and *Map7d2* depletion was associated with altered MAPK signaling, BMSCs were transfected with siRNAs targeting *GR*, *Map7d2*, or both simultaneously, followed by osteogenic induction (**Figure 7A**). Because MAPK-related processes were enriched in both RNA-seq datasets (**Figures 2**, **4**), we assessed Erk signaling. In two independent biological experiments, western blot analysis consistently showed that *GR* knockdown reduced Erk2 phosphorylation without affecting total Erk2 protein expression, whereas *Map7d2* knockdown increased Erk2 phosphorylation (**Figure 7B**). Combined depletion of *GR* and *Map7d2* restored Erk2 phosphorylation compared with *GR* depletion alone (**Figure 7B**). In contrast, Erk1 phosphorylation and total Erk1 protein expression exhibited only modest changes among the experimental groups (**Figure 7B**). Consistent with the changes in Erk2 phosphorylation, Alp protein expression was markedly reduced following *GR* depletion and was restored by co-transfection with si*Map7d2* (**Figure 7B**). Together, these results show a reproducible association between the partial rescue following combined *GR* and *Map7d2* depletion and the restoration of Erk2 phosphorylation and Alp protein expression. Based on these results, we propose a hypothesis-generating working model illustrating the potential relationships among *GR*, *Map7d2*, Erk2 signaling, and osteogenic differentiation (**Figure 7C**). The potential interactions are represented by dotted lines to distinguish them from experimentally established relationships.

**Figure 7:**
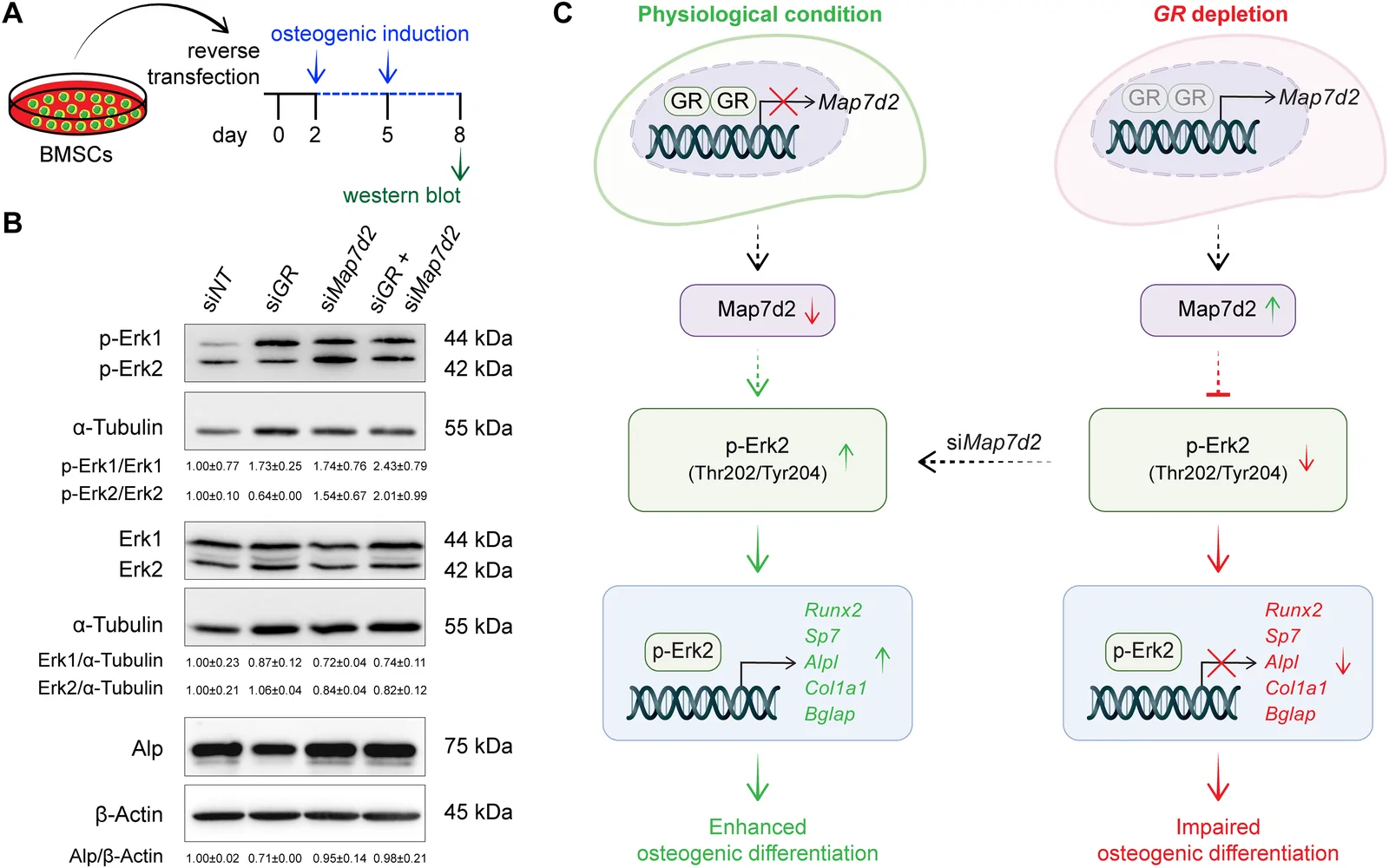
*GR* and *Map7d2* depletion is associated with altered Erk2 signaling in BMSCs. **(A)** Schematic overview of siRNA-mediated knockdown of *GR* and *Map7d2*, individually or in combination, in BMSCs. **(B)** Western blot analysis of phosphorylated Erk1 and Erk2 (p-Erk1/p-Erk2, Thr202/Tyr204), total Erk1 and Erk2, and Alp protein levels at day 8 post-transfection. Band intensities were quantified using ImageJ. Erk1 and Erk2 phosphorylation was quantified relative to the corresponding total Erk1 and Erk2 protein levels, respectively (n=2). **(C)** Proposed working model illustrating the potential relationship among GR, Map7d2, Erk signaling and osteogenic differentiation. The potential interactions among GR, Map7d2, and Erk2 are depicted using dotted lines.

## 4 Discussion

The present study confirms the importance of GR in regulating osteogenic differentiation and identifies a previously unrecognized association among *GR*, *Map7d2*, and Erk2 signaling. Using two independent primary murine osteogenic cell models, BMSCs and bone-derived primary osteoblasts, we show that *GR* depletion consistently impaired osteogenic differentiation despite eliciting distinct transcriptional responses in the two systems. Nevertheless, pathway enrichment analyses revealed shared biological processes, particularly MAPK signaling and cell migration. The requirement for physiological *GR* signaling during osteoblast differentiation is well established (24). Osteoblast-specific disruption of *GR* signaling impairs osteoblast maturation and bone formation, indicating that endogenous *GR* activity is essential for normal skeletal homeostasis and does not merely mediate the pathological effects of GCs (24). Consistent with these observations, *GR* depletion impaired osteogenic differentiation in both BMSCs and primary osteoblasts in our study. Our work extends these findings by characterizing the transcriptional changes induced by *GR* depletion and identifying GR-responsive candidates associated with osteogenic differentiation.

Interestingly, GR depletion had opposing effects in the two primary osteogenic cell models. Whereas BMSCs showed increased cell viability despite impaired osteogenic differentiation, bone-derived primary osteoblasts exhibited reduced viability accompanied by a broader suppression of osteogenic marker expression. These differences may reflect variation in cellular composition, tissue origin, isolation and culture conditions, or differentiation state, all of which may influence GR-dependent effects on proliferation and survival. Importantly, *GR* depletion impaired osteogenic differentiation in both models despite the opposing viability responses, indicating that the osteogenic phenotype cannot be explained solely by altered cell viability.

Comparative transcriptomic analysis showed that more than 2,700 genes were differentially expressed in both datasets, yet only 17 were regulated in the same direction. This marked divergence may reflect both biological and technical differences between the models. BMSCs and bone-derived primary osteoblasts differ in cellular composition, tissue origin, isolation and culture conditions, and transcriptional state. In addition, GR-dependent transcription is shaped by chromatin accessibility, lineage-associated transcription factors, and transcriptional co-regulators (19, 42). Thus, depletion of the same transcriptional regulator can produce substantially different gene-level responses in different cellular contexts. Nevertheless, pathway enrichment converged on shared processes, including MAPK signaling and cell migration. We therefore focused on concordantly regulated genes and shared pathways rather than assuming equivalent transcriptomic responses in the two models.

This systems-level interpretation also provides a rationale for focusing on common GR-responsive candidates rather than on individual differentially expressed genes. Among the few concordantly regulated genes in both osteogenic cell models, *Map7d2* emerged as a particularly compelling candidate because its role in skeletal biology has not previously been investigated. Although members of the *Map7* family are best known for their roles in microtubule organization and intracellular transport, growing evidence indicates that microtubule-associated proteins also influence cell polarity, migration, mechanotransduction, and signaling pathways essential for osteoblast function (43, 44). Our functional experiments showed that combined *GR* and *Map7d2* depletion partially restored cellular Alp activity and the expression of *Sp7*, *Alpl*, and *Bglap* compared with *GR* depletion alone, demonstrating a partial and marker-specific rescue of selected osteogenic readouts. However, this rescue was incomplete and marker-specific because *Runx2* and *Col1a1* expression was not significantly restored.

*Map7d2* depletion alone reduced cellular Alp activity despite increasing *Alpl* and *Bglap* mRNA expression, while *Runx2* expression was not changed. This discrepancy indicates that increased expression of selected osteogenic transcripts does not necessarily correspond to enhanced osteogenic differentiation or functional Alp activity. Cellular Alp activity does not necessarily directly reflect *Alpl* transcript abundance, as its function is additionally influenced by post-translational processing, including glycosylation, protein stability, membrane localization, and regulation of enzymatic activity (45). The increased expression of *Alpl* and *Bglap* following *Map7d2* depletion may therefore reflect a compensatory or dysregulated transcriptional response rather than enhanced osteogenic differentiation. Together, these findings indicate a context-dependent and complex association between *Map7d2* and osteogenic differentiation following *GR* depletion.

MAPK-associated pathways were among the shared biological processes in both osteogenic cell preparations. In two independent biological experiments, *GR* depletion was consistently associated with reduced Erk2 phosphorylation, whereas combined *GR* and *Map7d2* depletion was associated with restoration of Erk2 phosphorylation and Alp protein expression. These observations support an association between the partial rescue of selected osteogenic readouts and restoration of Erk2 phosphorylation. Erk signaling is a well-established regulator of osteoblast differentiation through its effects on osteogenic transcription factors and extracellular matrix production (16, 46). Although our findings indicate an association among GR, Map7d2, and Erk2 signaling, the molecular basis of these associations remains unresolved. The current data do not establish that GR directly regulates *Map7d2* transcription or whether Map7d2 directly controls Erk2 phosphorylation. Clarifying these relationships will require studies of GR occupancy at *Map7d2* regulatory regions and mechanistic experiments examining how *Map7d2* influences the Erk signaling pathway. Accordingly, **Figure 7C** presents a hypothesized working model. The potential interactions among GR, Map7d2, and Erk2 are depicted using dotted lines to indicate that they require further mechanistic validation.

Beyond osteoblast differentiation, our findings may have broader implications for osteoimmunology. Bone marrow stromal cells integrate endocrine, inflammatory, and mechanical cues to regulate bone remodeling and regeneration. As a ligand-activated transcription factor expressed in both immune and skeletal cells, *GR* occupies a central position at the interface between these signaling systems. The finding that *GR* depletion alters pathways associated with cell migration and MAPK signaling in osteogenic cells suggests that GR functions extend beyond regulation of classical osteogenic genes and may contribute to coordinating stromal responses within the bone marrow microenvironment. Future studies investigating how *GR*-dependent transcriptional programs influence immune-stromal communication during bone remodeling and fracture healing may therefore provide new insight into mechanisms linking skeletal regeneration with immune regulation (47–49).

Several limitations should be acknowledged. First, our conclusions are based on two *in vitro* primary murine cell models and require validation in conditional GR-deficient mice and human osteogenic cells. Second, transcriptomic analysis alone cannot define the chromatin-level mechanisms underlying the observed gene expression changes or establish *Map7d2* as a direct GR target. Studies of chromatin accessibility, transcription factor activity, GR occupancy, and primary transcriptional responses will be needed to address these questions. Finally, although the changes in Erk2 phosphorylation and Alp protein expression were reproducible in two independent biological experiments, the limited number of biological replicates precluded inferential statistical analysis. These data should therefore be interpreted descriptively.

In conclusion, our study demonstrates that GR is required for efficient osteogenic differentiation in two independent primary murine cell models and identifies common *GR*-dependent transcriptional and signaling responses across these systems. Our functional findings demonstrate a partial rescue of selected osteogenic readouts following combined *GR* and *Map7d2* depletion compared with GR depletion alone, whereas the western blot findings associate this partial rescue with restoration of Erk2 phosphorylation and Alp protein expression. Together, these findings identify *Map7d2* as a potential component of the GR-dependent regulatory network involved in osteogenic differentiation and provide a foundation for further investigation of the molecular mechanisms linking GR, Map7d2, and Erk signaling.

## Data Availability

The datasets presented in this study can be found in online repositories. The names of the repository/repositories and accession number(s) can be found in the article/**Supplementary Material**. The raw RNA-seq datasets discussed in this manuscript have been deposited in the NCBI's Gene Expression Omnibus (GEO) database (50) and are accessible through GEO Series accession number GSE346858.
